# Study on Chromium-Binding Capacity of *Callitriche cophocarpa* in an Aquatic Environment

**DOI:** 10.1007/s00244-012-9853-5

**Published:** 2012-12-18

**Authors:** Joanna Augustynowicz, Joanna Kyzioł-Komosińska, Sylwester Smoleń, Andrzej Waloszek

**Affiliations:** 1 Department of Botany and Plant Physiology, Faculty of Horticulture, University of Agriculture in Kraków, Al. 29 Listopada 54, 31-425 Kraków, Poland; 2 Institute of Environmental Engineering, Polish Academy of Sciences, ul. M. Skłodowskiej-Curie 34, 41-819 Zabrze, Poland; 3 Department of Soil Cultivation and Fertilization in Horticulture, Faculty of Horticulture, University of Agriculture in Kraków, Al. 29 Listopada 54, 31-425 Kraków, Poland; 4 Department of Plant Physiology and Biochemistry, Faculty of Biochemistry, Biophysics and Biotechnology, Jagiellonian University, ul. Gronostajowa 7, 30-387 Kraków, Poland

## Abstract

The aim of the present study was to investigate the binding strength of chromium (Cr) ions to aquatic macrophyte *Callitriche cophocarpa*. Shoots of the plants were incubated in a natural water solution containing Cr(III) or Cr(VI) at a concentration ranging from 0.5 to 4 mM under laboratory conditions. We found that *C. cophocarpa* has an extremely high capacity to bind Cr. The average level of accumulation reached 28,385 or 7,315 mg kg^−1^ dry weight for plants incubated with Cr(III) or Cr(VI), respectively. Shoots incubated in a 0.5 mM concentration of Cr(III) for 5 days removed almost 100 % of the metal from solution. The major pool of the bound Cr(III) ions follows the strongest mechanism of metal-binding to an organic matter. In contrast, we found that only 25 % of Cr(VI) ions are bound into the metallo-organic compounds and 57 % of Cr(VI) exists in an easily remobilizable form. Activity of a photosynthetic electron transport (as *F*_V_/*F*_M_) was evaluated with respect to the Cr-binding mechanism. Our results contribute to the development of knowledge on processes controlling bioremediation of heavy-metallic compounds in aquatic systems.

Chromium (Cr) is the sixth most abundant transition metal on Earth. When it is naturally incorporated into minerals, it exhibits a low chemical mobility and transport during erosion. However, the natural background of Cr is dwarfed by anthropogenic contamination. Global discharge of Cr is higher than those of lead (Pb), mercury (Hg), and cadmium (Cd) (Kabata-Pendias and Mukherjee 2007). Cr and Cr compounds are used in a wide variety of industrial and manufacturing applications, including steel alloy fabrication, plated product fabrication, nonferrous alloy metal fabrication, textile industry (for dyeing and printing), leather industry (for tanning), and manufacture of green varnishes, inks, paints, and glazes as well as in ceramics. In water and wastewater, Cr exists primarily in two oxidation forms: Cr(VI) and Cr(III). Cr(III) dominates as soluble Cr^3+^ at pH < 3. As pH increases, Cr(III) hydrolyzes to CrOH^2+^, Cr(OH)_2_^+^, Cr(OH)_3_, and Cr(OH)_4_^−^. In slightly acidic to alkaline conditions (pH 6–12) Cr(III) can precipitate as an amorphous Cr(OH)_3_. Depending on pH (6–8) and the concentration, aqueous solutions of Cr(VI) mainly contain dichromate (Cr_2_O_7_^2−^) or chromate (CrO_4_^2−^) anions. The Cr(VI) ions exert very toxic effects on living organisms. The high toxicity is due to a high redox potential of Cr(VI), its mobility, and its ability to penetrate biological membranes. In contrast, Cr(III) is an essential microelement in the mammalian metabolism. The role of Cr(III) in plant physiology has not yet been elucidated (Zayed and Terry 2003; Kabata-Pendias and Mukherjee 2007). Both, Cr(III) and Cr(VI) species exist in aquatic systems. The concentration of Cr(III) in water is lower than the concentration of Cr(VI) because Cr(III) has an ability to form complexes with organic ligands. The equilibrium between these two forms of Cr depends on many factors. These factors include pH, concentration of reducers (Fe(II), organic compounds), oxidizing mediators (O_2_, manganese oxides) and complexing agents (organic matter) (Kotaś and Stasicka 2000).

Common physicochemical treatment technologies that remove Cr pollution are based on reduction–oxidation, precipitation, accumulation, and sorption (Hawley et al. 2004). Phytoremediation belongs to a group of biological treatment technologies. It is based on the use of plants to eliminate a contaminant from the surrounding environment. Plants pose mechanisms of contaminant remediation related to the all of the mentioned strategies; however, the details of these mechanisms are not clear. A great deal of research has been conducted on phytoextraction, which is defined as an ability of a plant to remove and sequester a pollutant in its body (Prasad 2004).

An excellent review describing different types of adsorbents used in removal of Cr(III) and Cr(VI) from water is provided by Mohan and Pittman (2006). The materials—such as activated carbons, ion-exchange resins, and polysaccharide-based materials—are commercially used as sorbents. Disadvantages of using the above-mentioned materials include high operational costs and problems with the regeneration process. Therefore, the new low-cost adsorbents that demonstrate a high sorption capacity are clearly needed. Alive or dried microorganisms, fungi, algae, and higher plants are promising biosorbents of Cr. Studies of organic and inorganic materials for their applicability as sorbents in the removal of heavy metal ions from water and wastewater must not only consider the sorption capacity of the investigated materials but also the mechanisms by which metals are bound. The forms and types of the bindings are crucial for the mobility of a metal as well as its remobilization and bioavailability. A proper treatment of waste sorbents, i.e., recovery of the metal or its disposal, depend on the type of metal binding. Sequential extraction (SE) (Tessier et al. 1979) can identify main binding sites and quantify the strength of a metal binding to particulates. Different methods of chemical SE are used to fractionate metals in various environmentally reactive or hydromorphic phases of soil (Jaradat et al. 2006; Jain et al. 2008; Romaguera et al. 2008), sediment (Alomary and Belhadj 2007) and solid wastes (Ayari et al. 2008; Soco and Kalembkiewicz 2009). These chemical SE methods can differentiate between the following fractions: metals retained in a pore solution and adsorbed onto surface of particles, exchangeable species, metals bound to carbonates or manganese (Mn) and iron (Fe) oxides, metals bound to an organic matter and sulphides, and a residual phase of metals bound in lithogenic minerals. SE may also be applied to homogenous materials, e.g., organic matter, such as peat and plants, e.g., mosses (Mouvet and Claveri 1999; Kyzioł-Komosińska et al. 2006).

*Callitriche cophocarpa* (water starwort), the widely abundant aquatic macrophyte, was recently shown to remediate Cr(VI) in water (Augustynowicz et al. 2010). Compared with other aquatic and terrestrial accumulators that use root systems to accumulate (rhizofiltrate) metal ions, *C. cophocarpa* is capable to uptake Cr by shoot tissues. We previously showed that after incubation with 50 μM (2.6 mg L^−1^) Cr(VI), *C. cophocarpa* can accumulate >1,000 mg Cr kg^−1^ dry weight (dw) while maintaining its physiological status (Augustynowicz et al. 2010).

The aim of the present study was to determine whether *C. cophocarpa* can be used as an efficient biosorbent of dissolved, highly concentrated up to 4 mM (210 mg L^−1^) Cr(III) and Cr(VI) ions in a contaminated aquatic environment. In our experiments, concentrations of metals were chosen according to the Cr(III) and Cr(VI) content in electroplating wastewater and used in the adsorption tests (Tessier et al. 1979; Kyzioł-Komosińska and Kukułka 2008). We used a live plant material to mimic conditions that occur in a natural environment. During experiments, the physiological status of shoots was measured by evaluation of the photosynthetic light phase activity (as photosystem [PSII] activity by *F*_V_/*F*_M_ [see Maxwell and Johnson 2000; Strasser et al. 2004]) in relation to Cr remediation by the plant tissue. To measure the strength of Cr binding to *C. cophocarpa*, we used the SE method developed earlier by Tessier et al. (1979) and modified by Kersten and Förstner (1990). This technique is based on fractionation of Cr(III) and Cr(VI) ions in respect to their susceptibility to remobilization.

## Materials and Methods

### Plant Material

*Callitriche cophocarpa* Sendt. was collected in the Dłubnia River in southern Poland 50°16′ N/19°56′ E from February to November 2010. The material was thoroughly washed with tap water. Only healthy, mature shoots, approximately 10 cm in length, were separated and used for further experiments.

### Incubation of Plants in Cr Solutions

The Cr solutions were prepared using filtrated (pore size 0.4 μm; Millipore) river water derived from the natural nonpolluted *C. cophocarpa* environment. Water analysis was performed after inductively coupled plasma (ICP)–mass spectrometry spectroscopy (ELAN 6100; Perkin Elmer) (PN-EN ISO 9963-1:^2001^) as well as titration methods (PN-ISO 9297:^1994^; PN-EN ISO 17294-1:^2007^). The spectrometer was calibrated using the ICP multielement standard (Merck). The average amount (mg L^−1^) of elements were as follows: 10.07 Na^+^, 4.61 K^+^, 111.70 Ca^2+^, 13.71 Mg^2+^, 5 × 10^−3^ Fe^2+^, 7 × 10^−3^ Mn^2+^, 1.8 × 10^−2^ Zn^2+^, 2 × 10^−3^ Cu^2+^, 10^−3^ Mo^6+^, 32.40 Cl^−^, 32.90 SO_4_^2−^, 344.00 HCO_3_^2−^, 8.20 NO_3_^2−^, 0.06 PO_4_^3−^, and 0.20 BO_3_^3−^. The level of heavy metals Pb, Hg, Cd, and Cr did not exceed 1 μg L^−1^. The electrical conductivity of water was equal to 0.63 mS cm^−1^, pH = 7.80, and *E*_h_ (redox potential) = 256 mV. Cr was added as K_2_Cr_2_O_7_ in the case of Cr(VI) or Cr_2_(SO_4_)_3_·18H_2_O in the case of Cr(III) (POCH, Poland). The Cr salts were dissolved to obtain the final concentrations of 0.5 mM (26 mg L^−1^), 1 mM (52 mg L^−1^), 1.5 mM (78 mg L^−1^), 2 mM (104 mg L^−1^), and 4 mM (208 mg L^−1^) Cr. pH of the solutions decreased after addition of Cr salts. The starting pH of Cr(VI) was correlated with the Cr concentration and varied from 7.12 to 6.30. To prevent the precipitation of minerals, the pH of the water was decreased to 5.5 before application of Cr(III). Consequently, the pH values of the Cr(III) solutions were in the range of 4.90–4.54. The amount of Cr added (mM) was not significant in relation to the molar concentration of the intracellular solution, which usually is equal to a few hundred milimoles/liter. The shoots (3.5 g) were incubated in 150 ml Cr solution. The tests were performed for 5 days accordingly to previously published data (Kyzioł-Komosińska and Kukułka 2008). Incubations were conducted under controlled light and temperature conditions: 16 h, 35 μmol photons m^−2^ s^−1^ (LF 36W/54, Piła, Poland), 8 h darkness, at 25 °C. The light intensity was comparable with that of the natural *Callitriche* environment. During the experiments, the cultures were slightly rotary-shaken to imitate river flow and to improve element exchange. As a control, plants were incubated in identical conditions but without addition of Cr.

### Determination of Cr Content

After incubation, the plant material was thoroughly washed three times with distilled water and then dried for 24 h at 105 °C. The samples were then mineralized in a heated nitric acid (POCH, Toruń, Poland) and finally diluted to 10 ml. ICP–optical emission spectroscopy (Prodigy Teledyne Leeman Labs) was used to measure the total Cr content in the mineralized plant material as well as the Cr solutions. The spectrometer was calibrated using the ICP single-element standard (Merck). Analysis was performed at 205.552 nm.

### SE

The Cr ions were fractionated with respect to the major mechanisms of their sorption to *C. cophocarpa* but mainly with respect to the binding strength and susceptibility to remobilization. A classical seven-step SE scheme developed by Tessier et al. (1979) and modified by Kersten and Förstner (1990) was used. The fractionating procedure consisted of a subsequent treatment of the Cr-containing organic matter with extractants of increasing strength. This procedure enables to distinguish the metal enrichment “pools” F0 through F6 of increasing binding strength and decreasing mobility as follows:

- F0 = the pool of Cr ions occurring in the free-water space of the cell wall (the water-soluble fraction).
- F1 and F2 = the pools of Cr ions bound by electrostatic hydrogen and Van der Waals’ attraction between Cr ions and the surface charged cell wall or/and by exchange ion process between Cr ions and cations (K^+^, Ca^2+^, Mg^2+^, Fe^2+^) located in functional groups occurring on the surface of plant biosorbent walls (e.g., carboxyl, phenol hydroxyl) instead of H^+^ ions (mobile fractions).
- F3 and F4 = the pools of Cr ions trapped by chelation/complexation process of the surface functional groups (e.g., carboxyl, hydroxyl phenol, amine) into Cr–organic acid complexes (mobilizable fractions).
- F5 = the pool of very strong stable binding of Cr ions; Cr ions may be transported across the cell membrane and are located within the cell (according to accumulation process).
- Residual pool F6 = metals that are stably incorporated into the lithogenic crystallites of the mineral fraction. F6 does not occur in pure organic matter.

Plant samples were incubated in a solution containing 4 mM Cr. The samples (0.5 g) were then dried, homogenized, and analyzed by SE. Each of the above-described six fractions were centrifuged (20 min at 10,100×*g*) to separate the solid phase. In all extracts, Cr content was determined using a Thermo Jarrell Ash model AA-Scan-1 atomic absorption spectrophotometer equipped with Cr hollow cathode lamp under optimized measurement conditions. The spectrometer was calibrated using a standard Cr solution (Merck). The signal was measured with background correction (deuterium lamp) at the optical flame (A-Ac) height. The Cr content in soluble fraction (F0) from samples incubated in media consisting of Cr(VI) was determined using the UV–Vis method (*λ* = 546 nm; Varian Cary 50 Scan spectrophotometer) with 1,5**-**diphenylocarbazide in the acid medium.

### Measurement of Chlorophyll Fluorescence

Kinetics of the induction of chlorophyll fluorescence was recorded using Handy-PEA (Hansatech, UK) employing a chlorophyll fluorometer using the standard procedure. Fluorescence was induced by the red light: *λ*_max_ = 650 nm, 1,500 μmol photons m^−2^ s^−1^. Samples of leaves were preadapted in darkness for 30 min on wet filter paper rings. Recorded curves were then analyzed using the software PEA-Plus (provided by Hansatech, UK), and photosynthetic functional parameter *F*_V_/*F*_M_—the maximum potential quantum yield of PSII was calculated.

### Statistics

Up to five independent experiments were performed for each measurement. The results were analyzed using either a one-way or two-way analysis of variance (ANOVA) (*P* ≤ 0.05), and means were compared using Tukey’s test.

## Results

### Cr Binding: Accumulation Study

**Fig. 1:**
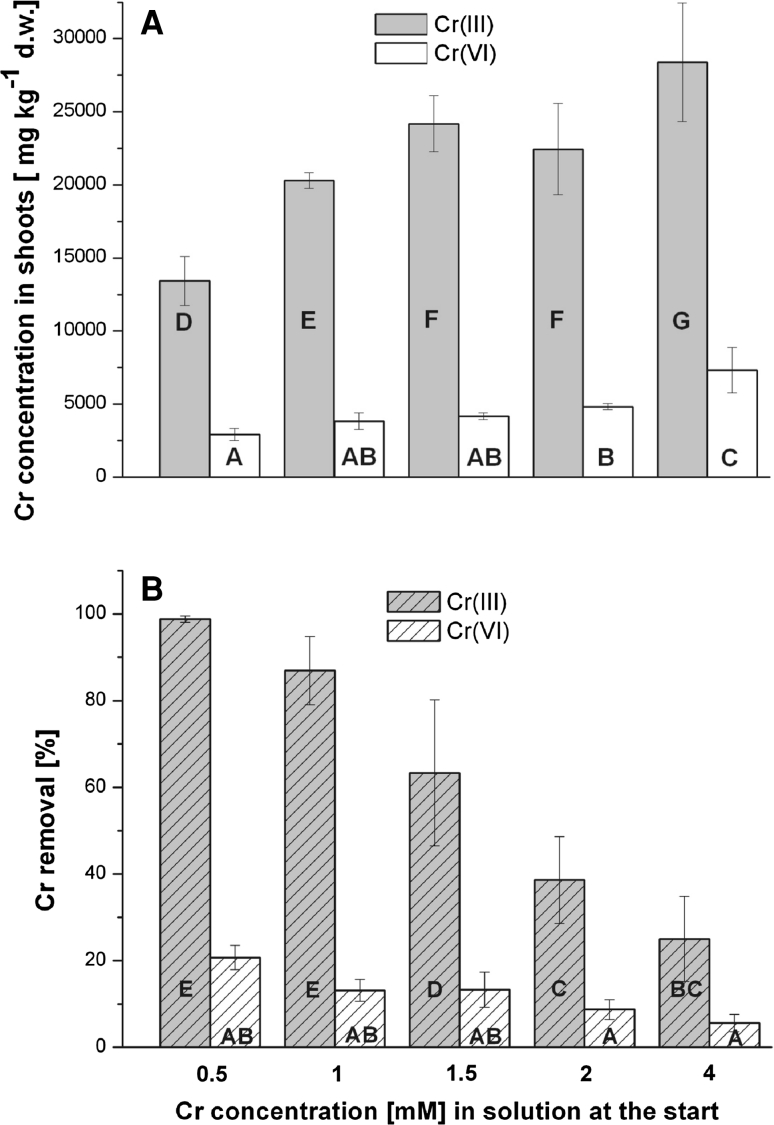
Accumulation (mg kg^−1^ dw) (**a**) and removal (%) (**b**) of Cr ions by shoots of *C. cophocarpa* during a 5-day incubation in relation to different Cr concentrations (mM) in medium. Different *letters* denote statistically significant differences (ANOVA and Tukey’s test; *P* ≤ 0.05). *Error bars* represent SDs. Each value is an average of six independent measurements

The accumulation of Cr ions by shoots was proportional to the applied concentration of the metal (Fig. 1a). However, the accumulation levels detected for 1.5 and 2 mM data points were not significantly different. Significant differences in Cr accumulation by shoots were found when 4 mM concentrations of Cr were present in solution. The amount of accumulated metal strongly depended on its speciation. The average accumulation level of Cr during the 5-day exposure was 4.8 times greater in the case of plants incubated in Cr(III) solution than in those incubated in Cr(VI). The highest metal content (28,385 mg kg^−1^ dw) was found in plants exposed to 4 mM Cr(III). Under the same condition, plants incubated in Cr(VI) solution accumulated up to 7,315 mg kg^−1^ dw. The level of Cr in control plants did not exceed 5 mg kg^−1^ dw. The accumulation of Cr by plants was correlated with the decrease of Cr content in the incubation medium (Fig. 1b). The statistical analysis showed differences in the correlation between Cr removal and the initial concentration of metal in solution for Cr(III) and Cr(VI). This correlation was linear in the case of Cr(III). In contrast, the removal of Cr(VI) was constant at all tested concentrations (compare homogenous groups). The most pronounced removal of Cr was measured in the case of Cr(III); ≤98.8 % of the Cr ions were removed from the solution after a 5-day treatment of the shoots. Efficiency of the Cr(VI) removal from the solution was, on average, 5.1 times lower. The concentration factor defined as Cr concentration in shoots (mg g^−1^ dw) versus Cr concentration in solution (mg g^−1^) ranged from 517 to 35 depending on the metal valence and its initial concentration (Table 1).

**Table 1:** Bioconcentration factor of Cr defined as a ratio of the metal concentration in shoots (mg kg^−1^ dw) to the concentration of the element dissolved in the solution (mg kg^−1^)

Initial Cr concentration (mM)	Concentration factor	
	Cr(III)	Cr(VI)
0.5	517	112
1	390	74
1.5	310	53
2	216	46
4	136	35

The pH of the solutions increased during the experiment in the all tested conditions, except from the media including the highest Cr(III) content, which are known to be strong acids. The increase of pH in the case of the Cr(VI) solution was similar irrespective of its initial concentration and gained 0.83. The pH of Cr(III) media increased in the range from 0.13 to 1.41. The decrease of pH (0.42 of pH units) was found in the highest Cr(III) concentration. No color changes were observed, and there was no precipitation in the Cr(III) solutions. No color change was detected in the Cr(VI) solution either. However, some precipitation in the 1–4 mM Cr(VI) concentrations were observed.

### Cr Binding: SE

**Fig. 2:**
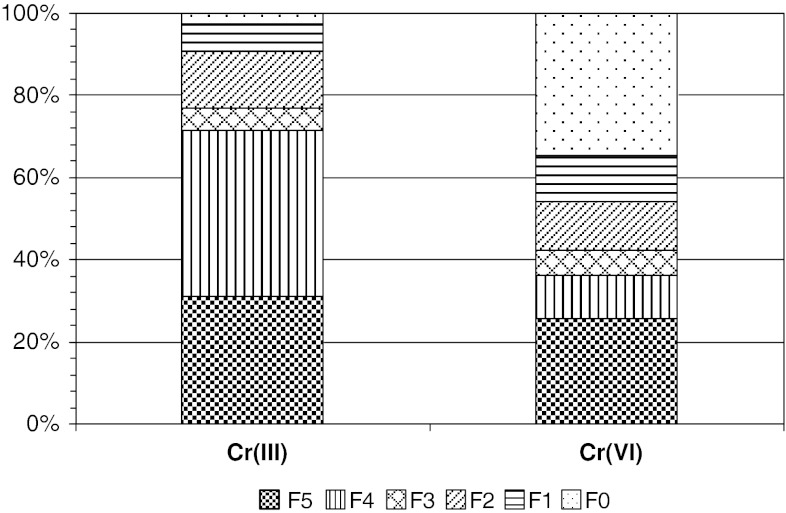
Sorption pools of different binding strength for Cr(III) and Cr(VI) binding onto *C. cophocarpa* after incubation of plants in 4 mM Cr concentration. *F0* pool of Cr ions occurring in the free-water space of the cell wall (the water-soluble fraction), *F1* and *F2* pools of Cr ions bound by the electrostatic, hydrogen, and Van der Waals’ attraction (mobile fractions), *F3* and *F4* pools of Cr ions trapped by chelation/complexation process (mobilizable fractions), *F5* pool of very strong, stable binding of Cr ions. Three independent measurements were performed for each sample

SE analysis showed a significant diversity in the main binding mechanism of Cr adsorbed to the studied plant material (organic matter). Examination of Cr binding strength after incubation of plants in Cr(III) solution showed that Cr was present in strong stable fractions (F5 and F4). In the mobile fractions (F0 and F1), Cr occurrence was small, i.e., 2.54 and 6.73 %, respectively (Fig. 2). The presence of Cr in F0 and F1 is important because these fractions show Cr that can be remobilized in various environmental conditions. Particularly, the highest relative amount of the metal adsorbed was in F4 (40.52 %) and F5 (30.99 %). The remaining fractions, F3 and F2, contained 5.45 and 13.77 %, respectively, of the total absorbed load (Table 2).

**Table 2:** Chemical fractionation (mg kg^−1^ dw) of natural organic matter of *C. cophocarpa* after incubation of plants in 4 mM Cr concentration

Chemical fractionation	F0		F1		F2		F3		F4		F5		ΣF0-F5	
	Mean	SD	Mean	SD	Mean	SD	Mean	SD	Mean	SD	Mean	SD	Mean	SD
Cr(III)	748	0.48	1975	0.46	4043	1.01	1614	1.67	11876	1.27	9100	2.50	29356	7.40
Cr(VI)	2689	3.76	896	0.80	909	1.13	481	1.78	831	0.84	1990	3.66	7795	8.40

*F0* pool of Cr ions occurring in the free-water space of the cell wall (the water-soluble fraction), *F1* and *F2* pools of Cr ions bound by the electrostatic, hydrogen, and Van der Waals’ attraction (mobile fractions), *F3* and *F4* pools of Cr ions trapped by chelation/complexation process (mobilizable fractions), *F5* pool of very strong stable binding of Cr ions. Three independent measurements were performed for each sample; SD (%)

Fractionation of the adsorbed Cr after incubation of the material in Cr(VI) media showed different distribution of Cr among the pools. In general, binding of the metal was significantly weaker (Fig. 2). Labile fraction F0 and F1 comprised 34.45 and 11.50 %, respectively, of the total adsorbed load, whereas stable F5 and F4 comprised 25.47 and 10.66 %, respectively. Moreover, the fractions F2 and F3 contained, as in the case of Cr(III), 11.65 and 6.26 % of the total adsorbed load, respectively (Table 2). The concentration of Cr(VI) ions in the water extract of the sample (F0) indicated the sole occurrence of Cr(VI). According to UV–Vis spectroscopy, the average level of Cr(VI) was 2,670 mg kg^−1^. A similar level of Cr(VI) was determined by atomic absorption spectrophotometry. The slight differences in the measured content of bound Cr likely reflect the different number of replicates we used for the assays.

### Photosynthetic Activity

#### *F*_V_/*F*_M_

**Fig. 3:**
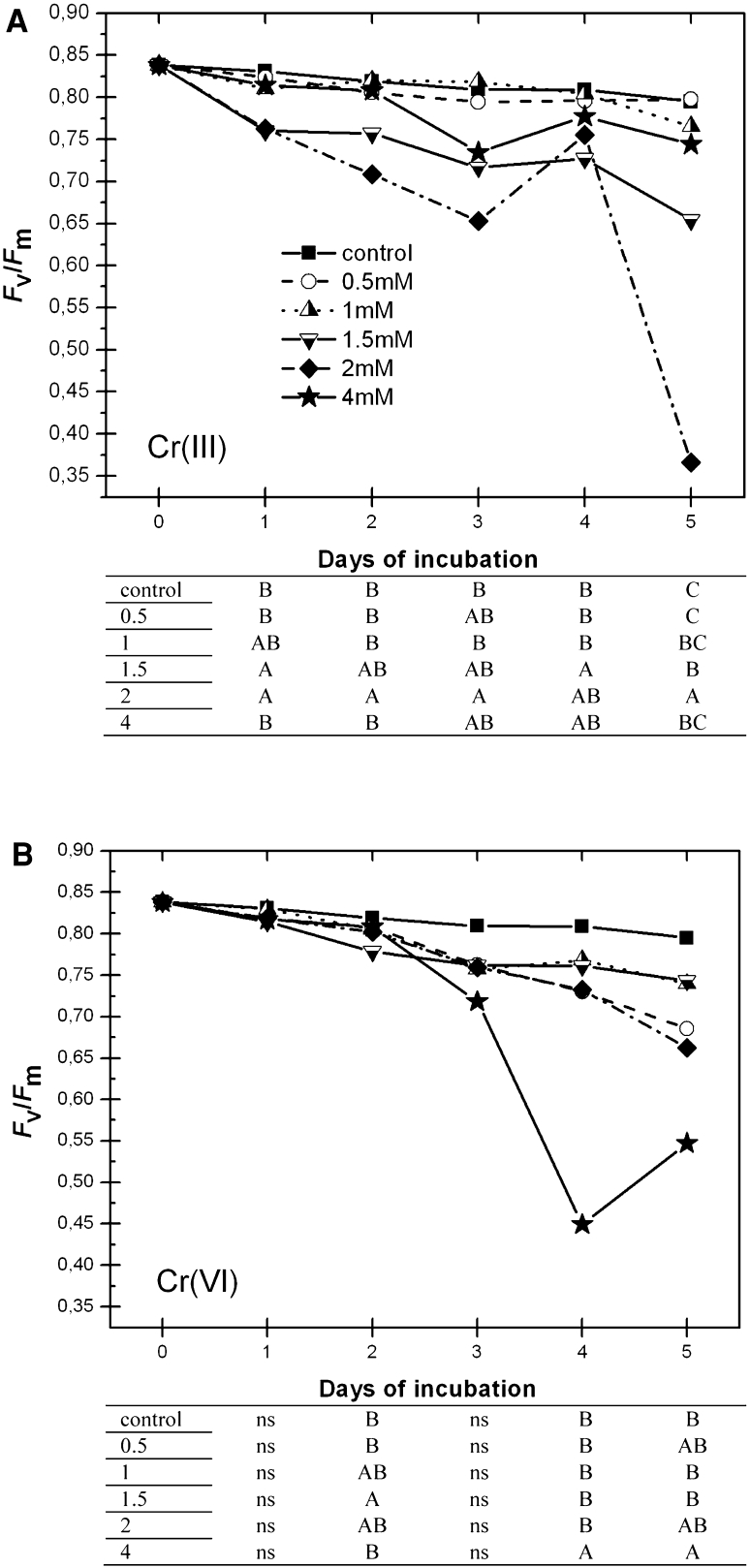
Maximum quantum yield of PSII (*F*_V_/*F*_M_) in leaves exposed to different Cr(III) and Cr(VI) concentrations during a 5-day incubation. Ten independent replicates were performed for each measurement. The *columns* of *tables* correspond to the days of incubation, and different *letters* denote statistically significant differences (ANOVA and Tukey’s test; *P* ≤ 0.05); *ns* not significant

The physiological parameter as counted out from chlorophyll fluorescence induction measurements is widely interpreted as a potential efficiency of photosynthetic energy conversion in PSII (Strasser et al. 2000). This parameter was not significantly influenced by the presence of 0.5 and 1 mM Cr(III) during 5 days of plant incubation (Fig. 3a). The Cr(III) concentrations of 1.5 mM, and especially 2 mM, caused a significant decrease in the PSII activity/photosynthetic electron flow in PSII. The highest tested concentration of Cr(III) (4 mM) inhibited photosynthesis starting from the day 3 of incubation, but the level of energy conversion was greater than that prompted by 1.5 and 2 mM Cr concentrations. Statistical analysis showed that *F*_V_/*F*_M_ was influenced by Cr(VI) on the days 2, 4, and 5 of the incubation (Fig. 3b). On day 2, the level of PSII activity was significantly decreased only in the sample containing 1.5 mM Cr(VI). The 0.5 mM Cr(VI) concentration caused a much stronger final effect on photosynthetic electron transport than Cr(III) at the same concentration. Surprisingly, on day 5 of incubation, the lowest Cr(VI) concentration exhibited an effect similar to the 2 mM concentration of Cr(VI). Results obtained for 1, 1.5, and 2 mM Cr(VI) were similar in the days following. The most negative influence of Cr(VI) on PSII activity was observed in the case of 4 mM Cr(VI).

## Discussion

Pollution with Cr compounds is a very serious environmental problem worldwide, affecting both the developed (e.g., the USA, where it is regarded as a priority contaminant by the United States Environmental Protection Agency) and developing (e.g., India) countries. Phytoremediation of water performed by vascular plants (macrophytes) is a competitive strategy compared with other bioremediation methods using prokaryotic and eukaryotic organisms, such as bacteria, unicellular algae, and yeast. Benefits arising from the use of vascular plants are related mainly to the ease of collecting material and no risk of migration of organisms in the soil–water environment. High phytoremediation potential of water macrophytes, especially occurring in hot climate zones, has been well documented in the literature (Dhote and Dixit 2009); however, the data concerning Cr are relatively scarce compared with that of other metal elements.

The idea of the present work was to find out, under laboratory conditions, whether *C.* *cophocarpa* could be an effective biofilter of the dissolved and highly-concentrated Cr(III) and Cr(VI) ions. The plant material was not grown in polluted environment, but it was moved to the contaminated water in the laboratory conditions to elucidate its potential biofiltering efficiency. Therefore, a live plant material and the water from the natural plant stand (filtrated to limit growth of algae and bacteria) was used for the experiments instead of the synthetic media. The pH of the Cr(VI) solutions was not manipulated. The pH of Cr(III) media had to be decreased to prevent chemical Cr(III) precipitation. The study also determined the binding strength of Cr onto the plant material and that is crucial for further appropriate use of this species as in low-cost treatment technologies. In the work reported here, we focused on the environmental significance of *C. cophocarpa* by testing it under semi-natural conditions.

*Callitriche cophocarpa* has been recently identified as a promising phytoremediator of low-concentrated Cr(VI) in aquatic systems (Augustynowicz et al. 2010). In the current study, high concentrations of both Cr oxidation states (Cr(III) and Cr(VI)) were used. In relation to other heavy metal aquatic phytoremediators (Zayed and Terry 2003), the average level of Cr accumulation by the shoots of *Callitriche* was extremely high, reaching almost 3 and 0.73 % dw, respectively, in the case of plant material incubated in Cr(III) and Cr(VI). Very significant values of Cr removal capacity and bioconcentration factors were also found. The above-mentioned parameters were comparable with those found in Cr aquatic hyperaccumulators as recently discovered *Leersia hexandra*. The greatest bioaccumulation coefficients of Cr in leaves of *Leersia* is 487 for Cr(III) and 72 for Cr(VI) (Zhang et al. 2007). Our results confirm the data reported earlier and indicate that aquatic macrophytes can accumulate more Cr(III) than Cr(VI) (Zayed and Terry 2003).

Uptake of Cr by plants is dependent on the oxidation state of the metal. In water systems under *E*_h_ ranging from 0 to 1,200 mV and pH < 5.0 (conditions like the ones of our experiment), the main Cr(III) species include cations Cr^3+^ and CrOH^2+^. At pH values greater than 13.5 and *E*_h_ ranging from 50 to −800 mV, soluble Cr(III) anions are formed (CrO^2−^). In contrast, under the experimental conditions used in this study (pH from 6.0 to 14.0, *E*_h_ from −100 to 900 mV), Cr(VI) forms soluble chromate (CrO_4_^2−^) anions (Brookins 1987). Consequently, distinct mechanisms are engaged in Cr binding to plants. Cr(III) accumulation is a passive process performed in two steps. The first step is based on the diffusion of Cr(III) ions across the cell wall and plasma membranes. This step is then followed by sorption of the cations onto the plant cell walls by way of different mechanisms that include (1) an electrostatic, hydrogen, and Van der Waals’ interaction between Cr ions and surface wall, (2) the chelation/complexes reaction between Cr ions and different chemical groups, e.g., hydroxyl, carboxyl, amide, sulfhydryl from the plasmalemma/cell wall, and (3) the exchange ion reaction between Cr and cations located in the surface functional groups instead of H^+^ (Kotaś and Stasicka 2000; Mohan and Pittman 2006). Compared with Cr(III) uptake, Cr(VI) anions are actively transported into the plant cells under control of sulphate transporters (Kaszycki et al. 2005; Appenroth et al. 2008). Passive sorption of Cr(VI) ions to the plant surface is far less pronounced than Cr(III) due to the differences in structure and toxicity of both ions. However, Cr(VI) could be reduced to Cr(III) by plants followed by sorption of the ions to the plant cell surfaces, which is an important phytoremediation strategy (Zayed and Terry 2003; Zazo et al. 2008; Espinoza-Quiñones et al. 2009). The ability of *C. cophocarpa* to reduce Cr(VI) to Cr(III) was shown using electron paramagnetic resonance spectroscopy (EPR; L-band) (Augustynowicz et al. 2009). In addition, recent experiments (Augustynowicz et al. 2012) conducted under the same conditions as those described herein showed that no plant-induced Cr(VI) reduction accompanying Cr accumulation was observed in Cr(VI) solutions. However, EPR spectroscopy showed that a biphasic signal of Cr(V) attending Cr(VI) to Cr(III) reduction was detected inside plant tissue every day during the study. Our results showed that phytoextraction, but not phytostabilization, is the main strategy of Cr(VI) detoxification by *C. cophocarpa* in aquatic systems. Based on the pattern of mean values found in the removal test (Fig. 1b), we postulate that different mechanisms of Cr uptake are involved in cases of Cr(III) and Cr(VI).

The SE method used in the study reported here was chosen as a standard technique that is useful in measurement of the strength of metal binding to organic matter. Because the interactions of Cr ions with proteins and other cell components are known, other conditions of sample preparation, e.g., air- or freeze-drying of samples instead of temperature-drying them, will be tested in the near future. SE exhibited a distinct valence-dependent metal-binding strength to the shoots of *C. cophocarpa*. We showed that the major pool of bound Cr(III) ions follows the strongest mechanism of metal binding to the organic matter, and the pool was located within the cell in the form of the chelate/complexes with surface organic groups. The results of fractionation of the Cr ions adsorbed by the plant after sample incubation in Cr(III) solution are consistent with the results of the chemical extraction from materials rich in organic matter (Kyzioł-Komosińska et al. 2006) and from the use of heterogenic materials (Romaguera et al. 2008; Lesven et al. 2009). In the present study, we have shown that there is no risk for secondary contamination of the aquatic environment by Cr discharging after saturation of *Callitriche* sorption capacity by Cr(III) ions. In contrast, cautious treatment of plant material accompanying Cr(VI) sorption is suggested. Although approximately 25 % of total Cr was also very strongly incorporated into plant tissue, a high amount of Cr was weakly bound to organic matter, 34 % in water soluble fraction and the next 23 % as easily mobile fractions. The analysis confirmed that only Cr(VI) ions, but not Cr(III) ions, are present in water extract of samples incubated in Cr(VI). Therefore, only the Cr(VI) fraction is susceptible to an easy release. To prevent contamination of soil and water with the desorbed Cr(VI) ions, the plant material, which was previously saturated with Cr(VI) should be stored in an isolated dry place until its disposal. The best way to dispose of the waste organic sorbent should include thermal treatment (incineration).

To evaluate the mechanism of physicochemical or metabolically derived Cr uptake by *Callitriche*, we studied the status of PSII activity, which is known to be very sensitive to the presence of heavy metals. Chlorophyll a fluorescence induction curve analysis was performed. This technique has been proven to be fast, nondestructive, and useful for studying the effects of different abiotic stresses on plants (Myśliwa-Kurdziel et al. 2004). According to the extremely high unphysiological concentration of Cr used in the present study, we had assumed physicochemical Cr binding by the plant under these conditions. However, taking into consideration the status of plants measured by way of efficiency of the photosynthetic energy conversion, a contribution of passive or active Cr transport and/or metabolic processes in Cr accumulation by the species in these highly concentrated metal solutions cannot be excluded. Changes of the *F*_V_/*F*_M_ parameter shows that action of both Cr(III) and Cr(VI) on *Callitriche* plants cause a decrease in the photosynthetic activity, but the metabolism of the cells remain still active. The effect of both Cr forms on *Callitriche* is not strictly concentration dependent. Cr(III) ions at 1.5 and 2 mM concentrations showed far stronger effects than that of 4 mM. Furthermore, 0.5 mM Cr(VI) caused an *F*_V_/*F*_M_ decrease comparable with that of 2 mM and much greater that that of 1 and 1.5 mM. This observation is not consistent with our earlier observation concerning the action of a lower Cr(VI) concentration (Augustynowicz et al. 2010) as well as the one reported by other investigators (Hörcsik et al. 2007; Oláh et al. 2010). In the previously mentioned works, direct dependence of Cr(VI) on *F*_V_/*F*_M_ was measured. There are possibly two explanations for this phenomenon. First, our earlier work was performed in synthetic media with lower pH, which is known to favor higher redox activity of Cr(VI) (Kotaś and Stasicka 2000). Consequently, the negative influence of Cr(VI) on plant metabolism, following the decrease in efficiency of photosynthetic energy conversion in PSII, is more pronounced in media with lower pH. Second, we can speculate that different resistance mechanisms may be active in plants at the applied Cr concentration. Another evidence of possible active metabolism contribution in Cr transport in *Callitriche* might be a result of pH change. We speculate that the pH increase in Cr(VI) solutions could be an effect of metabolically derived transport of Cr(VI) anions (e.g., Cr_2_O_7_^2−^) that coincide with the efflux of OH^−^ (HCO_3_^−^). The increase in pH value of Cr(VI)-enriched nutrient media was also observed by Espinoza-Quiñones et al. (2009) in the study of *Eichhornia crassipes*, *Salvinia auriculata*, and *Pistia stratiotes*. In contrast, the pH increase in the Cr(III) media (Cr_2_(SO_4_)_3_) might be triggered by preferential uptake of sulphate anions by plants instead of Cr(III) cations.

In the group of Cr adsorbents currently available, the most effective ones are activated carbons and ion-exchange resins. In the case of activated carbons, the calculated maximal Cr-adsorption capacities, depending on the type of material used, is in the range of approximately 0.15–30 % (Mohan and Pittman 2006). However, high operational costs related to the costs of the material production and reactivation are the main disadvantages in their use. Therefore, naturally derived, low-cost sorbents, including zeolites, clay minerals, and oxides together with biosorbent, such as microorganism, algae, mosses, and higher plants are tested (Hawley et al. 2004; Mohan and Pittman 2006). According to the results of statistical analysis and by comparing the homogenous groups related to accumulation of Cr, we assume that the maximal sorption capacity of Cr by *C. cophocarpa* has not yet been determined. Hence, our next study will focus on the exact calculation of the Cr accumulation capacity of *Callitriche* based on the adsorption models. For that reason, the Langmuir or Freundlich models could be used. It would allow evaluation of the maximal Cr sorption capacity of *C. cophocarpa* and compare it to other sorbents applied in Cr removal. However, it must be pointed out that calculated adsorption properties are usually not environmentally relevant due to the specific conditions used during the incubation procedure, such as very low pH or high temperature. Still, the extraordinary accumulation capacity of Cr ions by *C. cophocarpa*, found in the conditions reported in this study, is comparable with the ones described for commercially used sorbents.

In conclusion, *C. cophocarpa* was identified as an effective biofilter of dissolved Cr ions in a contaminated aquatic environment. Our results indicate that this plant can be used as an efficient, low-cost Cr biosorbent for further use in wastewater purification technologies.
